# The Atrial Fibrillation Health Literacy Information Technology Trial: Pilot Trial of a Mobile Health App for Atrial Fibrillation

**DOI:** 10.2196/17162

**Published:** 2020-09-04

**Authors:** Emily Guhl, Andrew D Althouse, Alexandra M Pusateri, Everlyne Kimani, Michael K Paasche-Orlow, Timothy W Bickmore, Jared W Magnani

**Affiliations:** 1 Heart and Vascular Institute University of Pittsburgh Medical Center Pittsburgh, PA United States; 2 Department of Medicine University of Pittsburgh School of Medicine Pittsburgh, PA United States; 3 School of Medicine University of Pittsburgh Pittsburgh, PA United States; 4 College of Computer and Information Science Northeastern University Boston, MA United States; 5 Section of General Internal Medicine Department of Medicine Boston University Boston, MA United States

**Keywords:** atrial fibrillation, health-related quality of life, medication adherence, health literacy, mobile phone

## Abstract

**Background:**

Atrial fibrillation (AF) is a common arrhythmia that adversely affects health-related quality of life (HRQoL). We conducted a pilot trial of individuals with AF using a smartphone to provide a relational agent as well as rhythm monitoring. We employed our pilot to measure acceptability and adherence and to assess its effectiveness in improving HRQoL and adherence.

**Objective:**

This study aims to measure acceptability and adherence and to assess its effectiveness to improve HRQoL and adherence.

**Methods:**

Participants were recruited from ambulatory clinics and randomized to a 30-day intervention or usual care. We collected baseline characteristics and conducted baseline and 30-day assessments of HRQoL using the Atrial Fibrillation Effect on Quality of Life (AFEQT) measure and self-reported adherence to anticoagulation. The intervention consisted of a smartphone-based relational agent, which simulates face-to-face counseling and delivered content on AF education, adherence, and symptom monitoring with prompted rhythm monitoring. We compared differences in AFEQT and adherence at 30 days, adjusted for baseline values. We quantified participants’ use and acceptability of the intervention.

**Results:**

A total of 120 participants were recruited and randomized (59 to control and 61 to intervention) to the pilot trial (mean age 72.1 years, SD 9.10; 62/120, 51.7% women). The control group had a 95% follow-up, and the intervention group had a 93% follow-up. The intervention group demonstrated significantly higher improvement in total AFEQT scores (adjusted mean difference 4.5; 95% CI 0.6-8.3; *P*=.03) and in daily activity (adjusted mean difference 7.1; 95% CI 1.8-12.4; *P*=.009) compared with the control between baseline and 30 days. The intervention group showed significantly improved self-reported adherence to anticoagulation therapy at 30 days (intervention 3.5%; control 23.2%; adjusted difference 16.6%; 95% CI 2.8%-30.4%; *P*<.001). Qualitative assessments of acceptability identified that participants found the relational agent useful, informative, and trustworthy.

**Conclusions:**

Individuals randomized to a 30-day smartphone intervention with a relational agent and rhythm monitoring showed significant improvement in HRQoL and adherence. Participants had favorable acceptability of the intervention with both objective use and qualitative assessments of acceptability.

## Introduction

### Background

Atrial fibrillation (AF) is a highly prevalent cardiac arrhythmia. AF is challenging for patients because it typically requires long-term adherence to anticoagulation for stroke prevention, symptom assessment, symptom monitoring, and navigating subspecialty care [1]. AF is an important cause of stroke, heart failure, and death. In addition, the symptoms, treatment burden, prognostic uncertainty, and adverse effects on general health and functional status associated with AF may worsen health-related quality of life (HRQoL) in patients with the condition [2]. The effect of AF on HRQoL may be amplified by limited health literacy [3], which can exacerbate the challenges patients face in negotiating a chronic and complex disease such as AF. Individuals with limited health literacy are particularly vulnerable to AF as the condition requires education, decision-making, and long-term adherence. Previous work looking at one-time educational sessions in those with limited health literacy and AF did not improve outcomes in AF [3]. In a population where limited health literacy has an impact on patient-centered outcomes, an intervention that allows for ongoing intervention, such as a mobile app, may improve outcomes.

### Objectives

**Figure 1:**
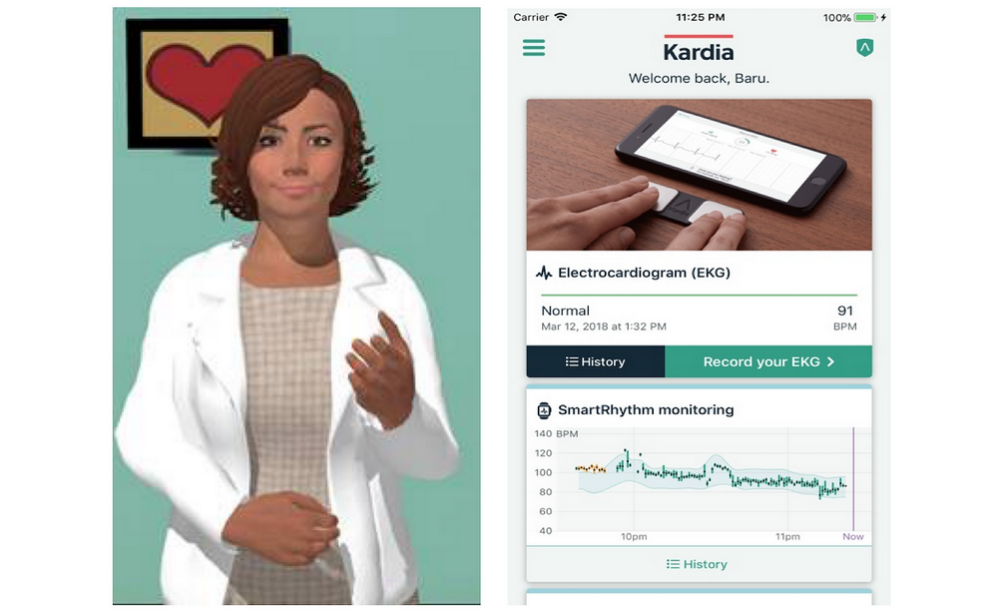
Relational agent as presented by a smartphone to individuals randomized to the intervention (left); Kardia app as presented by a smartphone (right).

We have developed a mobile health technology intervention using a relational agent with the goal of improving patient-centered care in AF [4]. The agent functions by simulating a face-to-face conversation with a health counselor using synthetic speech and an animated counselor that uses nonverbal conversational behaviors such as hand gestures and facial displays (Figure 1). In each interaction, the relational agent dialogue is tailored with the patient using their name and personal information as well as responding to their conversational inputs from the current and prior conversations. Our team has used relational agents in multiple health contexts with the goal of fostering a therapeutic alliance and assisting self-care in individuals with chronic medical conditions, particularly those with limited health literacy [5-9]. We have implemented relational agents to improve self-care and health outcomes such as increasing physical activity in older adults, improving communication at hospital discharge to prevent readmission rates, and educate patients for shared decision-making [5,10-12]. Relational agents provide an interactive resource for longitudinal patient engagement that contrasts with traditional media for patient education, such as web-based videos or literature. Relational agents have the further advantage of expressing empathy during interactions in addition to the opportunities to conduct didactic interventions, repeated assessments, and monitoring.

This pilot trial implemented a limited relational agent designed to improve adherence and HRQoL in individuals with AF. In conjunction with the relational agent, we used AliveCor’s Kardia mobile heart rate and rhythm monitor (Kardia), a validated and Food and Drug Administration–approved instrument for smartphone-based heart monitoring [13]. Our relational agent curriculum taught patients how to use and interpret the Kardia and guided Kardia use when they reported symptoms. We expect that the real-time feedback of the Kardia-recorded rhythm, particularly when individuals are experiencing symptoms, will enhance patient understanding of the disease. We hypothesized that our intervention would result in better AF-specific HRQoL and adherence when compared with usual care in a pilot cohort of 120 participants.

## Methods

### Trial Design and Recruitment

We conducted a single-center, parallel arm pilot trial, termed the Atrial Fibrillation Health Literacy Information Technology Trial, registered at ClinicalTrials.gov (NCT030935558). Our trial was conducted according to the CONSORT (Consolidated Standards of Reporting Trials) statement and guidelines for pilot and feasibility trials. Our primary outcome for this study was HRQoL measured with the Atrial Fibrillation Effect on Quality of Life (AFEQT) measure (range 0-100, higher scores associated with superior HRQoL). Our secondary outcomes included self-reported adherence to anticoagulation and assessment of intervention acceptability by qualitative and quantitative measurements.

Study participants were recruited while receiving care at ambulatory facilities at the University of Pittsburgh Medical Center, a large regional health care system spanning multiple sites in Pittsburgh, Pennsylvania. Participants were identified by reviewing the electronic medical record, referral to the study using the University of Pittsburgh’s Center for Assistance in Research eRecord protocol, referral by clinical providers, or participant-initiated self-referral. Inclusion criteria were (1) age ≥18 years, (2) history of chronic AF, (3) prescribed oral anticoagulation for stroke prevention secondary to AF, and (4) English-speaking sufficient to use a smartphone-based relational agent as ascertained by the study screener. Participants were excluded from this pilot trial if they had AF deemed attributable to a non-cardiac cause, had undergone cardiothoracic or thoracic surgery within 30 days of evaluation, were unable to use the smartphone apps after training, had a life expectancy of <12 months as identified by a concurrent diagnosis (such as malignancy), or by determination of the research team for not being able to participate in the informed consent process. Prior experience with a smartphone was not a criterion for participation. Individuals without a smartphone randomized to the intervention arm were provided with one for their general use during the 30-day study period. Participants in the intervention were also given the Kardia device. Intervention participants received a training session on how to use the smartphone, relational agent, and Kardia apps. Participants randomized to the intervention were provided with instructions on how to access the relational agent and the Kardia. They were provided with a comprehensive orientation to the phone and the intervention apps. Participants were provided with instructions until they demonstrated adequate familiarity with the instruments. All participants recorded an electrocardiogram with the Kardia under study personnel supervision. The orientation session was concluded when participants were able to demonstrate how to turn the phone on, charge it, and access and use the apps. The study protocol and informed consent were reviewed and approved by the University of Pittsburgh Institutional Review Board.

### Patient and Public Involvement

Content for the relational agent was informed by interviewing individual patients about their experience of AF. During the interviews, patients identified the principal challenges of AF and self-management. Patients described obstacles to understanding the disease, its causes, chronicity, and potential outcomes; adherence to anticoagulation, including both intentional (eg, forgetting and electing to forego) and nonintentional (eg, transportation to pharmacy) adherence to anticoagulation; symptom recognition and how to respond to symptoms; self-care approaches to AF, such as monitoring symptoms; and preparation for the clinical encounter.

### Relational Agent Development and Content

The intervention arm includes a smartphone-based relational agent named *Tanya* (Figure 1) that simulates a face-to-face conversation with a health coach using synthetic speech and accompanying animated behavior such as hand gestures. *Tanya* functions to augment patient-centered health care by providing health education, monitoring, and problem-solving for users. The content is tailored for individual use by using the user’s name and appropriate time context (eg, “Good Afternoon”). As the user goes through the relational agent’s content, she is given the choice to select from a menu of responses, which then prompts the relational agent’s response. In addition, the relational agent can be programmed to refer to prior content areas and obtain repeated assessments to follow for the resolution of reported problems. The content for the relational agent was developed by a review of patient-centered domains, review of the literature, and qualitative interviews with patients. Prior work has demonstrated that relational agents provide health education and counseling that are accessible to individuals with limited health and computer literacy and diverse socioeconomic backgrounds [5-9]. We developed a dialogue for this relational agent by reviewing patient-centered domains, literature, and qualitative interviews with patients with AF. The dialogue content was organized as modules that focused on 3 different domains: AF education, symptoms, and adherence. Relational agent dialogue referred to the Kardia regularly to reinforce its use, provide instruction on the use of the device, and direct users to check rhythm concomitant with reporting symptoms. We monitored the modular content accessed and frequency and duration of relational agent usage.

### Participant Assessments

Assessments were obtained at clinical sites following the administration of informed consent. Participant age, sex, race, and ethnicity were obtained from electronic medical records. Smoking status, educational attainment, and annual income were self-reported. BMI was extracted from the medical records. Clinical history, including hypertension, diabetes, cardiovascular disease, heart failure, and prior stroke or transient ischemic attack, was obtained from the medical records, as were medications (ie, anticoagulants, antiarrhythmics, and rate control agents) and treatments (ie, cardioversion and pulmonary vein isolation) relevant to AF. For the measure of health literacy, we used the Short-Test Of Functional Health Literacy in Adults, which is a 36-item measure that is scored from 1 to 36, with higher scores indicating superior health literacy and scores of 23 indicating limited health literacy [14]. Depression was measured using the Patient Health Questionnaire-9, a validated 9-item questionnaire scored from 0 to 27, with scores >10 correlating with the presence of depression and increasing scores correlating with increasing depression severity [15].

We assessed HRQoL and self-reported anticoagulant adherence at baseline and at 30 days. The AFEQT is a validated 20-item instrument measuring self-reported HRQoL specific to AF (range 0-100, higher scores associated with superior HRQoL) [16]. In addition to providing a global measure of AF-specific HRQoL, the AFEQT measures the impact of AF on HRQoL across the 4 domains of symptoms, daily activities, treatment concerns, and treatment satisfaction. The overall AFEQT score is calculated using a composite of the first 3 domains: symptoms, daily activities, and treatment concerns. The domain scores are calculated using the sum of the responses of the answers for each specific category. Both composite and domain AFEQT scores range from 0 to 100, with higher scores representing superior HRQoL. Medication adherence was measured at enrollment and follow-up by asking participants about their specific agent for anticoagulation. Participants were asked (1) “Do you sometimes forget to take [name of prescribed anticoagulant medication]?” and (2) “Over the past two weeks, were there days that you did not take [name of prescribed anticoagulant medication]?” Participants randomized to the intervention arm completed assessments and interviews at 30 days to report and describe their response to the relational agent. Qualitative assessment of relational agents was performed by administering patient questionnaires with free-text responses to those in the intervention arm. Examples of questions included in the acceptability assessment were “What did you like least about the application?,” “What were your overall impressions of Tanya?,” or “How did you feel about having Tanya with you all of the time?.” Responses were recorded and assessed for representative quotations.

### Randomization and Data Collection

Individuals eligible for participation in the study were approached by the study team. After agreeing to participate and undergoing informed consent, they were randomized, with allocation concealed, 1:1 to receive the intervention or usual care using a computer-generated randomization scheme with the Research Electronic Data Capture hosted at the University of Pittsburgh [17]. Randomization was not blinded as individuals receiving the intervention underwent installation of the relational agent and Kardia apps on their smartphones or received a study smartphone with these apps for temporary use. Individuals in the intervention arm were instructed to use the apps daily. Study staff, outcome assessors, and data analysts were not blinded to the allocation as the intervention group had additional assessments of the app.

### Statistical Analysis

For our sample size calculation, we assumed an SD in the AFEQT score ranging from 16 to 24 units, consistent with prior literature and a smaller-sized, single-arm demonstration of our intervention [4,18,19]. With an estimated population mean difference of 12 points between the intervention and control arms and an SD of 22 points, a total sample size of 120 participants would have 85% power to show a difference between the intervention and control groups. Continuous variables were summarized by their mean and SD and categorical variables by their frequency and percentage. ANCOVA (analysis of covariance) was used to compare differences in follow-up AFEQT scores between the intervention and control arms adjusting for baseline scores [20]. A 2-tailed alpha value of .05 was deemed statistically significant. All analyses were conducted as intention-to-treat, with no participants excluded from analyses, regardless of their adherence to the intervention. As this study was a pilot trial, no adjustments were made for multiple comparisons. Statistical analyses were performed using SAS (version 9.4; SAS Institute).

## Results

### Baseline Characteristics

Multimedia Appendix 1 describes the enrollments and follow-up of the study. From July 2017 to April 2018, a total of 527 individuals eligible for participation were identified as attending scheduled clinic visits. Of these, the study team was able to approach 236, of whom 129 agreed to participate. The first available 120 individuals were then consented for enrollment and randomized (59 to the control group and 61 to the intervention group). Table 1 describes the characteristics of the 120 enrolled participants by the study arm. Participants were aged 72.1 (SD 9.1) years, and 51.7% (62/120) participants were women. Age and sex distributions were similar in the 2 arms, although control arm participants were more likely to have heart failure and diabetes than those in the intervention arm. The cohort was well educated, with 60.8% (73/120) having a bachelor’s degree or higher. Of the total cohort, 35.0% (42/120) reported an annual household income of <US $50,000. Of the 61 participants randomized to receive the intervention, 93.4% (57/61) completed the 30-day follow-up and 4 decided to leave the study. There were 59 individuals randomized to the control, of whom 94.9% (56/59) completed the 30-day follow-up, with the remaining leaving the study for unknown reasons.

**Table 1:** Characteristics of pilot trial participants by treatment arm.

Characteristics^a^		All participants (n=120)	Control (n=59)	Intervention (n=61)
Age (years), mean (SD)		72.1 (9.10)	72.6 (7.28)	71.7 (10.6)
Female gender, n (%)		62 (51.7)	30 (50.8)	32 (52.5)
White race, n (%)		111 (92.5)	54 (91.5)	57 (93.4)
BMI (m/kg^2^), mean (SD)		30.9 (6.79)	31.0 (5.92)	30.8 (7.61)
**Smoking history, n (%)**				
	Never	63 (52.5)	30 (50.8)	33 (54.1)
	Former	53 (44.1)	25 (42.4)	28 (45.9)
	Current	4 (3.3)	4 (6.8)	0 (0.0)
Heart failure, n (%)		24 (20.0)	14 (23.7)	10 (16.4)
Preserved, n (%)		15 (12.5)	9 (15.3)	6 (9.8)
Reduced, n (%)		9 (7.5)	5 (8.5)	4 (6.6)
Hypertension, n (%)		105 (87.5)	50 (84.7)	55 (90.2)
Diabetes, n (%)		29 (24.2)	17 (28.8)	12 (19.7)
Stroke/TIA^b^, n (%)		1 (0.8)	1 (1.7)	0 (0.0)
Vascular disease, n (%)		30 (25.0)	15 (25.4)	15 (24.6)
**Education, n (%)**				
	High school/vocational	34 (28.3)	21 (35.6)	13 (21.3)
	Some college	13 (10.8)	6 (10.2)	7 (11.5)
	Bachelor’s degree	33 (27.5)	17 (28.8)	16 (26.2)
	Graduate	40 (33.3)	15 (25.4)	25 (41.0)
**Income (US $), n (%)**				
	<19,999	10 (8.3)	6 (10.2)	4 (6.6)
	20,000-49,999	32 (26.7)	17 (28.8)	15 (24.6)
	50,000-99,999	30 (25.0)	13 (22.0)	17 (27.9)
	>100,000	25 (20.8)	11 (18.6)	14 (23.0)
S-TOFHLA^c^, mean (SD)		30.1 (4.5)	30.3 (4.0)	30.0 (4.9)
S-TOFHLA ≤23, n (%)		10 (8.3)	4 (6.8)	6 (9.8)
PHQ-9^d^ score, (units)		3 (1-4)	3 (1-6)	3 (1-4)

^a^Continuous variables are presented as mean (SD), and categorical variables are presented as number (percentage).

^b^TIA: transient ischemic attack.

^c^S-TOFHLA: Short-Test Of Functional Health Literacy in Adults.

^d^PHQ-9: Patient Health Questionnaire-9.

### AFEQT scores

Multimedia Appendix 2 graphically displays the AFEQT scores of the control and interventional arms at baseline and 30-day follow-up. Table 2 reports the between-group contrast in 30-day AFEQT scores by total score and individual domains with covariate adjustment for baseline scores. Intervention participants had better scores in total AFEQT (adjusted mean difference 4.5; 95% CI 0.6-8.3; *P*=.03) and daily activity domain (adjusted mean difference 7.1; 95% CI 1.8-12.4; *P*=.009) scores compared with the control with adjustment for baseline. Anticoagulant adherence data are presented in Table 3, which shows significantly greater improvement in the interventional group compared with the control group for both self-report anticoagulant adherence items.

**Table 2:** Atrial Fibrillation Effect on Quality of Life scores, baseline and 30-day follow-up, by treatment arm.

Scores and subscores	Control, mean (SD)		Intervention, mean (SD)		Adjusted mean difference		
	Baseline	Follow-up	Baseline	Follow-up		Adjusted mean difference (95% CI)^a^	*P* value^b^
AFEQT^c^ symptom	83.7 (19.7)	82.8 (21.2)	85.9 (14.5)	87.6 (15.2)		3.1 (−1.3 to 9.6)	.26
AFEQT daily activity	69.6 (23.8)	69.5 (22.3)	77.6 (19.9)	82.6 (18.6)		7.1 (1.8 to 12.4)	.009
AFEQT treatment	79.4 (20.1)	80.4 (21.2)	83.8 (15.7)	87.1 (14.8)		2.9 (−1.9 to 7.8)	.24
AFEQT satisfaction	79.3 (22.9)	79.3 (19.3)	78.5 (23.1)	83.3 (20.9)		4.3 (−2.6 to 11.3)	.22
AFEQT total	76.0 (17.6)	76.1 (16.7)	81.5 (14.2)	85.2 (14.1)		4.5 (0.6 to 8.3)	.03

^a^Estimate of the adjusted mean difference (95% CI) between follow-up AFEQT score in intervention group versus control group from analysis of covariance model with follow-up AFEQT score as outcome variable, adjusting for baseline score as covariate.

^b^*P* value from analysis of covariance model comparing follow-up AFEQT score between intervention group versus control group with adjustment for baseline score.

^c^AFEQT: Atrial Fibrillation Effect on Quality of Life.

**Table 3:** Self-reported adherence to anticoagulation by treatment arm.

Adherence questions	Control, n (%)		Intervention, n (%)			
	Baseline (n=59)	Follow-up (n=56)	Baseline (n=61)	Follow-up (n=57)	Adjusted % difference (95% CI)^a^	*P v*alue^b^
Do you sometimes forget to take (name of anticoagulant medication)?^c^	13 (22)	13 (23.2)	17 (27.9)	2 (3.5)	16.6 (2.8 to 30.4)	<.001
Over the past 2 weeks, were there any days you did not take (name of anticoagulant medication)?^c^	4 (6.8)	6 (10.7)	11 (18)	2 (3.5)	7.9 (−1.5 to 17.2)	.09

^a^Estimate of the adjusted percentage difference (95% CI) of follow-up adherence in the intervention group versus control group from logistic regression model with follow-up adherence as outcome variable, adjusting for baseline adherence as covariate.

^b^*P* value from logistic regression model comparing follow-up adherence between intervention group versus control group with adjustment for baseline adherence.

^c^Numbers and percentages reflect the number of participants answering “yes” to each item.

### Adherence

We observed moderate adherence to the intervention. Participants in the intervention had a median of 18 (interquartile range 19) conversations with the relational agent over 30 days and used the agent for a median of 15 (interquartile range 13) days. The mean total duration of interactions with the relational agents was 40.7 (SD 24.3) min over the 30-day period, averaging 2.1 (SD 1.0) min per conversation. Of the 61 participants in the intervention group, 48 (79%) completed the AF education module and 43 (70%) completed the medication adherence counseling module (Textbox 1). The number of symptoms reported to the relational agent ranged from 0 to 14 (mean 1.3, SD 2.3). The median number of days of Kardia use was 25 over the 30-day intervention with a range of 1 to 30 days of usage.

**Summary of relational agent domain content.**

**Education**

- Causes of atrial fibrillation
- Atrial fibrillation treatment strategies
- Stroke prevention in atrial fibrillation
- AliveCor Kardia use, troubleshooting

**Symptoms**

- Overview of common symptoms
- Chest pain and chest pressure
- Heart racing or palpitations
- Dyspnea and shortness of breath
- Fatigue

**Adherence**

- Overview of adherence
- Adherence to medications
- Adherence barriers
- Strategies to address barriers

**Patient activation**

- Goals for self-management
- Preparing for the medical encounter

### Acceptability

Multimedia Appendices 3 and 4 present representative examples of conversations that intervention participants had with the relational agent. Multimedia Appendix 3 presents an exchange that a user had describing a symptom and the use of the AliveCor Kardia heart rate and rhythm monitor to correlate symptomatic palpitations with cardiac rhythm assessment. Multimedia Appendix 4 illustrates the relational agent teaching a user about the relevance and utility of using the Kardia. Our qualitative assessments of acceptability informed us that most intervention participants found the relational agent useful, informative, and trustworthy. On a range from 1 (“not at all”) to 7 (“very satisfied”), intervention participants indicated a median score of 6 (response range 1 to 7), which indicated that they were satisfied working with the relational agent. Likewise, participants indicated that they found talking with the agent easy (median 1, response range 1 [“easy”] to 7 [“difficult”]). Participants reported that the content was repetitive with a median score of 5 (response range 1 to 7), with 1 indicating “not at all” and 7 indicating “very repetitious.” Direct quotes from intervention participants are that the relational agent “made it easier to accept the information” and that the app provided “control to do something simple daily to take care of myself in this stressful situation.” We summarize the selected quotes in Textbox 2.

**Representative responses of pilot trial participants randomized to the intervention arm.**

**What were your overall impressions of the atrial fibrillation app?**

- “You can bring up information on Afib on the internet and read most of the same stuff on your own, but with Tanya you have a structured presentation that you're led through, so you end up getting the information you should be getting.”

**What were your overall impressions of Tanya?**

- “I liked the fact that she didn't seem to make me afraid or say very drastic things.”
- “She made it easy to accept the information.”
- “I had good impressions.”
- “It's like going to the doctor’s office every day to learn about A fib.”

**What did you like most about the app?**

- “EKG is helping me figure out if I am in Afib and matching up the symptoms that I'm feeling with what that says can be helpful.”
- “You're going through what I'm going through, and you’re stressed out and depressed. At least each day I got a sense that I was trying to make myself better and do something for myself by talking to Tanya.”

**How did you feel about having Tanya with you all of the time?**

- “Feeling like I had control to do something simple daily to take care of myself in this stressful situation.”

**What did you like least about the app?**

- “Tanya was artificial, she wasn't real. But she was fun.”
- “It was hard for me to think of her as a real person.”
- “It was repetitive, nothing new after a while.”

## Discussion

### Principal Findings

In this pilot trial of 120 individuals with chronic AF, we implemented a novel intervention that combined a relational agent with the Kardia heart rate and rhythm monitor for smartphones. After 30 days, intervention participants reported significantly better HRQoL as measured by the AF-specific AFEQT measure than control participants, as well as better self-reported adherence to anticoagulation. Our results support that a relational agent in combination with the Kardia may have the potential to improve patient-reported outcomes in AF. These results extend our previous work, which has demonstrated that relational agents are an appropriate vehicle for patient education, monitoring, and problem-solving.

In this pilot trial, we focused on HRQoL and adherence to anticoagulation because of their importance for long-term success with this chronic condition. HRQoL is a central benchmark in AF management, as recognized by the United States and international professional society guidelines [1,21]. As a patient-centered outcome, HRQoL provides a meaningful gauge for how patients experience a chronic disease. Individuals with AF may have extensive symptoms and experience the burden of long-term treatment with increased risk for multiple adverse outcomes, all of which adversely affect HRQoL. We consider the AFEQT as an appropriate measure to evaluate HRQoL because of its specificity to AF and relevant domains (symptoms, daily activities, treatment concern, and treatment satisfaction). Adherence to anticoagulation is vital for the prevention of long-term cardioembolic stroke. Evidence suggests the challenges that patients have with maintaining adherence to anticoagulation therapy with either warfarin or direct-acting oral anticoagulants [22-24].

Participants receiving the intervention in this pilot trial had better AFEQT scores than those receiving the control after 30 days. The minimally important difference for change in the AFEQT score has been suggested as 12 units in a moderate-sized, 3-month study of patients undergoing electrophysiologic interventions for control of AF [25]. We used this quantity to determine the statistical power for this pilot trial. Although statistically significant, the magnitude of improvement for the total AFEQT score did not meet this threshold. However, we did observe that control participant scores were essentially unchanged between the baseline and 30-day assessments. Therefore, we conclude that the pilot was effective in demonstrating that a relational agent can improve HRQoL in individuals with AF. We are particularly encouraged by the improvement in adherence reported by the intervention participants, given the essential role of anticoagulation in stroke prevention and AF pharmacologic management. HRQoL warrants continued attention as a patient-reported outcome in AF. Most evaluation of HRQoL in AF is related to treatment pharmacologic and invasive therapies for the condition. We expect that enhanced patient education, symptom monitoring, and development of self-care skills—all appropriate for a relational agent curriculum—may improve the patient experience with AF and concomitantly enhance HRQoL and medication adherence.

In our pilot study, the limited improvement in AFEQT may have stemmed from multiple factors. First, the duration of the intervention was only 30 days, which we considered adequate for a pilot trial with limited relational agent content. We expect that a longer duration of use could bolster the effect of the relational agent on HRQoL. Second, more extensive relational agent content, both in terms of scope as well as the depth of exchanges, would improve the value of the relational agent to patients. Our content here—focused on education, anticoagulant adherence, and symptom identification—was of limited scope. Although we had good engagement, we expect that enhanced content has the potential to bolster sustained, longer-term use. A more expansive content may extend participant engagement and provide avenues for greater depth of self-care tasks, such as monitoring and responding to symptoms and adherence challenges. We surmise that both the duration and the content as employed in this pilot study were likely not adequate to result in a more meaningful improvement in HRQoL. Likewise, we encouraged participants to use the Kardia after reporting symptoms. However, we did not link relational agent content to those results to enhance the correlation of symptoms with rate and rhythm recordings. Finally, we did not link the intervention to avenues for the modification of patient care in this pilot study. The relational agent delivered via smartphones has the potential to monitor symptoms coupled with the Kardia results. Such monitoring may, in turn, provide important clinical information to support the adjustment of therapies for AF. For mobile health app content to have sustained impact, it must provide results to the hub of clinical care. We intend to address the deficits described here in subsequent apps of a relational agent for AF. Our objective is to develop a more extensive relational agent with a better interface with the Kardia results. In addition, we intend to build mechanisms for reporting the unique data obtained by the interventions to clinicians, thereby facilitating improvement to patient care.

### Strengths and Limitations

We successfully combined our smartphone relational agent and Kardia technologies and showed that this approach was highly acceptable and enhanced patient self-care for patients with AF. We found significant improvements in HRQoL and self-reported anticoagulant adherence in the intervention arm. Our results provide substantive data to guide an enhanced relational agent for a larger-sized trial and encourage the development of a more extensive relational agent.

Our study has several important limitations. First, the limited time frame and content may have reduced engagement with the intervention. Second, we did not assess the sustainability of the intervention effect. Third, this pilot cohort was predominantly White and relatively well educated. We have designed the relation agent to be accessible for people with limited health literacy; however, an examination of potential differential treatment effects will require a more diverse cohort. Fourth, we measured adherence using self-reported measures rather than an objective measure of adherence; the self-reported measure is subject to reporting and response-shift bias. Fifth, we did not have an active control group. As such, we are not able to distinguish the mechanism for improvements in our outcomes in the intervention arm of our pilot trial. Such improvements may be attributable to specific effects of the intervention’s content, the increased attention provided by receiving a relational agent, or the novelty of providing a smartphone and app. It is also possible that engagement with the agent was enhanced by participants’ awareness of use being monitored. Next, we also recognize that our study had a selection bias. We were only able to approach 236 of 527 eligible participants, noting that many who were eligible were not accessible to us (ie, did not attend clinic appointments where the study team was conducting recruitment). Additionally, given loss to follow-up, missing data, and study withdrawal, we did not achieve our calculated estimated sample size of n=120, which limits our power. We acknowledge this as a limitation because the final sample size was short of the planned 120 in our power calculation. Sixth, relational agent content was informed by interviewing patients about their challenges with AF. Although we did not conduct this assessment in a systematic manner, we considered the content for this pilot trial adequately informed by patient input. Finally, participant assessments were not conducted by blinded assessors, which may have introduced biases in responding to questions such as self-reported adherence.

This pilot trial provides the foundation for a larger clinical trial guided by these preliminary efficacy and acceptability results. Although we saw promising results in the pilot trial, our results reflect the effect of a small number of participants, particularly when evaluating adherence; a larger clinical trial will further evaluate and confirm this effect. We expect that the relational agent coupled with the Kardia has the potential to improve patient-centered care in AF and provide a low-cost, effective means of reducing the social and medical morbidity associated with this chronic disease.

### Conclusions

In this pilot trial (n=120) evaluating a novel, AF-focused relational agent in concert with the Kardia heart rhythm monitor, we found that individuals receiving the intervention had significantly greater improvement in AF-specific HRQoL and self-reported anticoagulant adherence. In addition, the relational agent demonstrated favorable acceptability with adequate usage in the intervention group and positive qualitative assessments of the app.

## Appendix

Multimedia Appendix 1 Flow diagram of progress through trial.

Multimedia Appendix 2 Comparison of Atrial Fibrillation Effect on Quality of Life scores, by study arm, at baseline and 30-day follow-up.

Multimedia Appendix 3 Naomi.

Multimedia Appendix 4 Frank.

## Abbreviations

AF: atrial fibrillation
AFEQT: Atrial Fibrillation Effect on Quality of Life
HRQoL: health-related quality of life

## References

[1] January CT, Wann LS, Alpert JS, Calkins H, Cigarroa JE, Cleveland JC, Conti JB, Ellinor PT, Ezekowitz MD, Field ME, Murray KT, Sacco RL, Stevenson WG, Tchou PJ, Tracy CM, Yancy CW, ACC/AHA Task Force Members (2014). 2014 AHA/ACC/HRS guideline for the management of patients with atrial fibrillation: executive summary: a report of the American college of cardiology/American heart association task force on practice guidelines and the heart rhythm society. Circulation.

[2] Dudink EA, Erküner O, Berg J, Nieuwlaat R, CB CB, Weijs B, A A, AJ AJ, Breithardt G, Le Heuzey JY, Luermans JG, Crijns HJ (2018). The influence of progression of atrial fibrillation on quality of life: a report from the Euro heart survey. Europace.

[3] Magnani JW, Mujahid MS, Aronow HD, Cené CW, Dickson VV, Havranek E, Morgenstern LB, Paasche-Orlow MK, Pollak A, Willey JZ, American Heart Association Council on Epidemiology and Prevention; Council on Cardiovascular Disease in the Young; Council on Cardiovascular and Stroke Nursing; Council on Peripheral Vascular Disease; Council on Quality of Care and Outcomes Research;Stroke Council (2018). Health literacy and cardiovascular disease: fundamental relevance to primary and secondary prevention: a scientific statement from the American Heart Association. Circulation.

[4] Magnani JW, Schlusser CL, Kimani E, Rollman BL, Paasche-Orlow MK, Bickmore TW (2017). The atrial fibrillation health literacy information technology system: pilot assessment. JMIR Cardio.

[5] Bickmore TW, Silliman RA, Nelson K, Cheng DM, Winter M, Henault L, Paasche-Orlow MK (2013). A randomized controlled trial of an automated exercise coach for older adults. J Am Geriatr Soc.

[6] Utami D, Bickmore TW, Barry B, Paasche-Orlow MK (2014). Health literacy and usability of clinical trial search engines. J Health Commun.

[7] Bickmore TW, Utami D, Matsuyama R, Paasche-Orlow MK (2016). Improving access to online health information with conversational agents: a randomized controlled experiment. J Med Internet Res.

[8] Gwynn KB, Winter MR, Cabral HJ, Wolf MS, Hanchate AD, Henault L, Waite K, Bickmore TW, Paasche-Orlow MK (2016). Racial disparities in patient activation: evaluating the mediating role of health literacy with path analyses. Patient Educ Couns.

[9] Zhang Z, Bickmore TW, Paasche-Orlow MK (2017). Perceived organizational affiliation and its effects on patient trust: role modeling with embodied conversational agents. Patient Educ Couns.

[10] King AC, Bickmore T, Campero I, Pruitt L, Yin L (2013). Employing virtual advisors in preventive care for underserved communities: results from the COMPASS study. J Health Commun.

[11] Bickmore TW, Pfeifer LM, Paasche-Orlow MK (2009). Using computer agents to explain medical documents to patients with low health literacy. Patient Educ Couns.

[12] Ehrenfeld J, Sandberg W, Kwo J, Bickmore T (2010). Use of a Computer Agent to Explain Anesthesia Concepts to Patients. Relational Agents Group.

[13] Halcox JP, Wareham K, Cardew A, Gilmore M, Barry JP, Phillips C, Gravenor MB (2017). Assessment of remote heart rhythm sampling using the Alivecor heart monitor to screen for atrial fibrillation: the REHEARSE-AF study. Circulation.

[14] Baker DW, Williams MV, Parker RM, Gazmararian JA, Nurss J (1999). Development of a brief test to measure functional health literacy. Patient Educ Couns.

[15] Kroenke K, Spitzer RL, Williams JB (2001). The PHQ-9: validity of a brief depression severity measure. J Gen Intern Med.

[16] Spertus J, Dorian P, Bubien R, Lewis S, Godejohn D, Reynolds MR, Lakkireddy DR, Wimmer AP, Bhandari A, Burk C (2011). Development and validation of the atrial fibrillation effect on quality-of-life (AFEQT) questionnaire in patients with atrial fibrillation. Circ Arrhythm Electrophysiol.

[17] Harris PA, Taylor R, Thielke R, Payne J, Gonzalez N, Conde JG (2009). Research electronic data capture (REDCap)--a metadata-driven methodology and workflow process for providing translational research informatics support. J Biomed Inform.

[18] Kotecha D, Ahmed A, Calvert M, Lencioni M, Terwee CB, Lane DA (2016). Patient-reported outcomes for quality of life assessment in atrial fibrillation: a systematic review of measurement properties. PLoS One.

[19] Randolph TC, Simon DN, Thomas L, Allen LA, Fonarow GC, Gersh BJ, Kowey PR, Reiffel JA, Naccarelli GV, Chan PS, Spertus JA, Peterson ED, Piccini JP, ORBIT AF Investigators and Patients (2016). Patient factors associated with quality of life in atrial fibrillation. Am Heart J.

[20] Harrell Jr FJ, Slaughter J (2001). Biostatistics for Biomedical Research. University School of Medicine Nashville.

[21] Kirchhof P, Benussi S, Kotecha D, Ahlsson A, Atar D, Casadei B, Castella M, Diener HC, Heidbuchel H, Hendriks J, Hindricks G, Manolis AS, Oldgren J, Popescu BA, Schotten U, van Putte B, Vardas P, Agewall S, Camm J, Esquivias GB, Budts W, Carerj S, Casselman F, Coca A, de Caterina R, Deftereos S, Dobrev D, Ferro JM, Filippatos G, Fitzsimons D, Gorenek B, Guenoun M, Hohnloser SH, Kolh P, Lip GY, Manolis A, McMurray J, Ponikowski P, Rosenhek R, Ruschitzka F, Savelieva I, Sharma S, Suwalski P, Tamargo JL, Taylor CJ, van Gelder IC, Voors AA, Windecker S, Zamorano JL, Zeppenfeld K (2016). 2016 ESC guidelines for the management of atrial fibrillation developed in collaboration with EACTS. Eur J Cardiothorac Surg.

[22] Brown JD, Shewale AR, Talbert JC (2016). Adherence to rivaroxaban, dabigatran, and apixaban for stroke prevention in incident, treatment-naïve nonvalvular atrial fibrillation. J Manag Care Spec Pharm.

[23] Beyer-Westendorf J, Ehlken B, Evers T (2016). Real-world persistence and adherence to oral anticoagulation for stroke risk reduction in patients with atrial fibrillation. Europace.

[24] Fang MC, Go AS, Chang Y, Borowsky LH, Pomernacki NK, Udaltsova N, Singer DE (2010). Warfarin discontinuation after starting warfarin for atrial fibrillation. Circ Cardiovasc Qual Outcomes.

[25] Dorian P, Burk C, Mullin CM, Bubien R, Godejohn D, Reynolds MR, Lakkireddy DR, Wimmer AP, Bhandari A, Spertus J (2013). Interpreting changes in quality of life in atrial fibrillation: how much change is meaningful?. Am Heart J.

