# Supplementary figures and images for: The Atrial Fibrillation Health Literacy Information Technology Trial: Pilot Trial of a Mobile Health App for Atrial Fibrillation
Source: JMIR Cardio. 2020 Sep 4;4(1):e17162. doi: 10.2196/17162 (PMC7501575; doi:10.2196/17162)

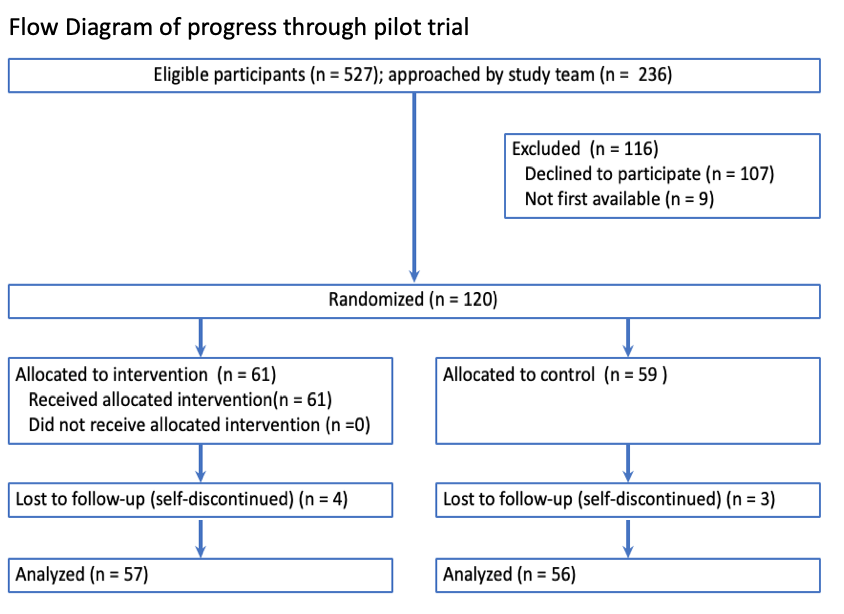

Supplement: Multimedia Appendix 1 [file cardio_v4i1e17162_app1.png]

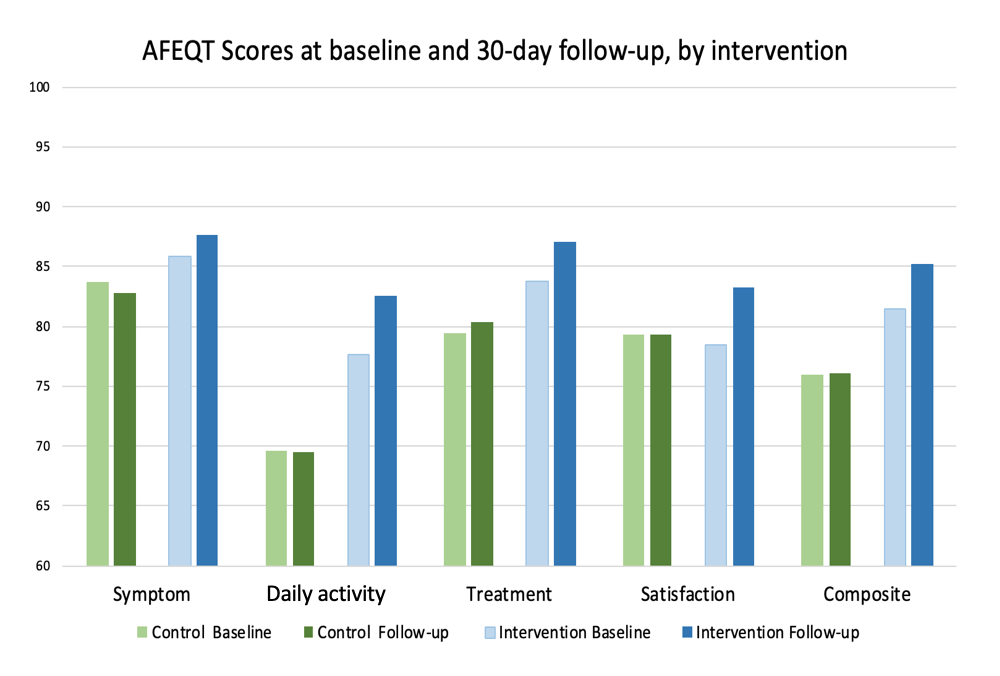

Supplement: Multimedia Appendix 2 [file cardio_v4i1e17162_app2.png]

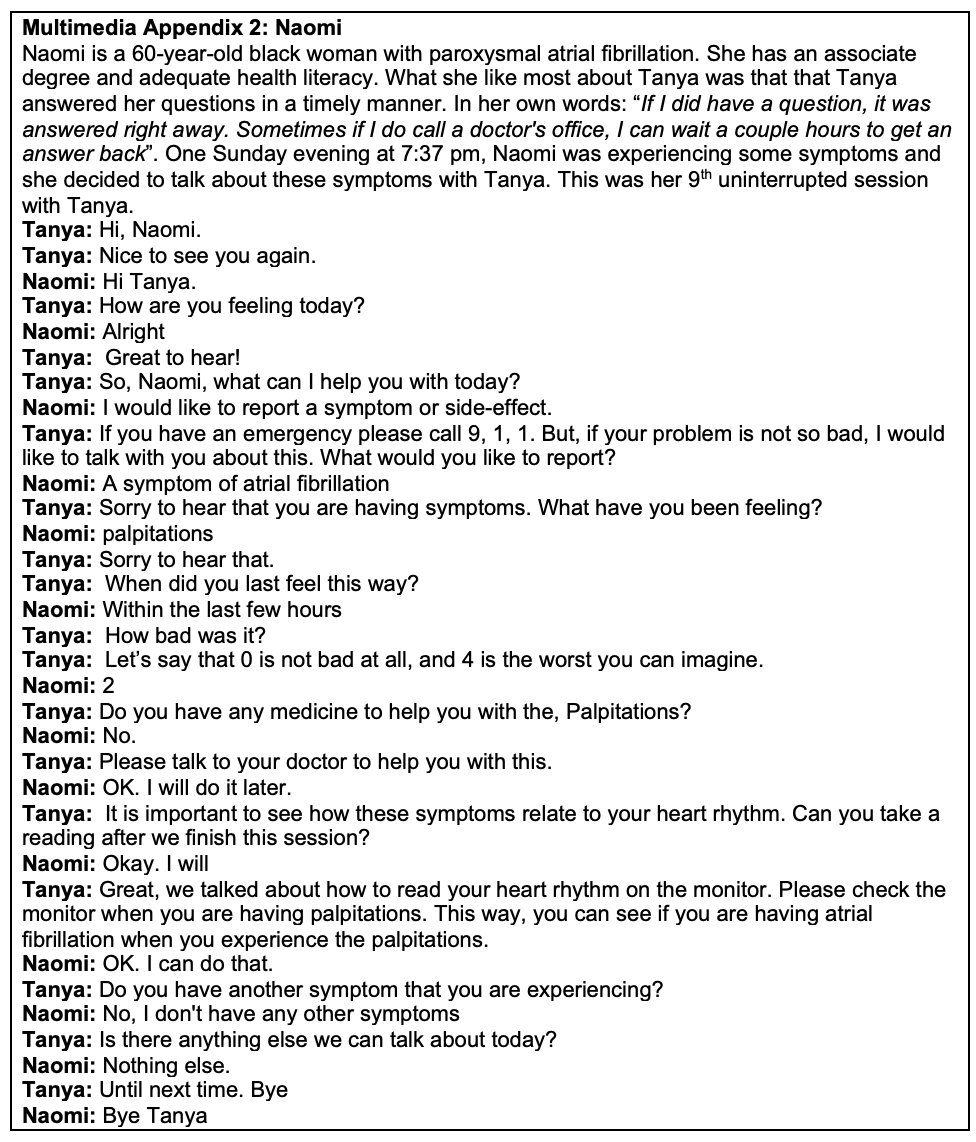

Supplement: Multimedia Appendix 3 [file cardio_v4i1e17162_app3.png]

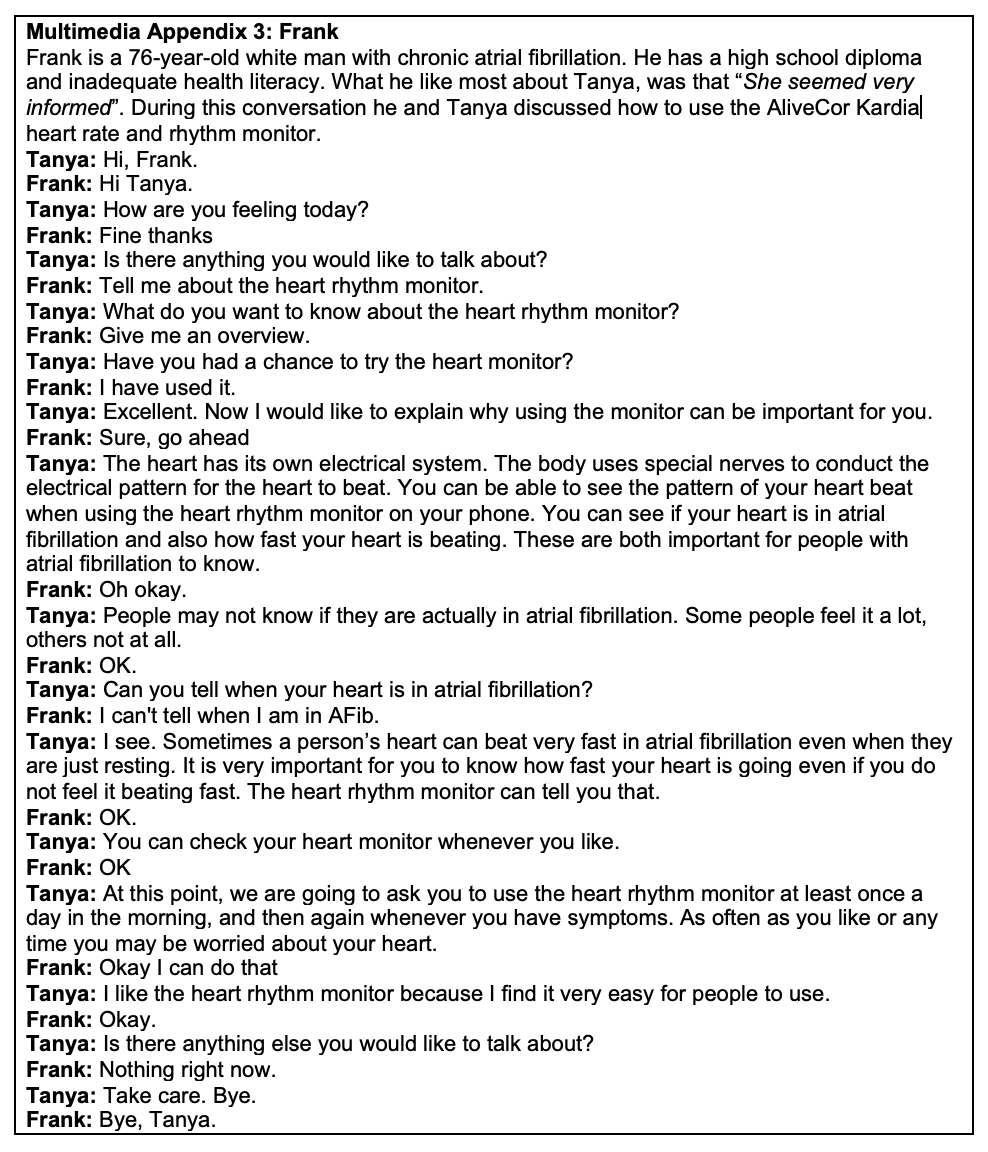

Supplement: Multimedia Appendix 4 [file cardio_v4i1e17162_app4.png]
